# Aerobic and Anaerobic Exercise’s Impact on Cognitive Functions in Eighth Grade Students

**DOI:** 10.3390/ijerph21070833

**Published:** 2024-06-26

**Authors:** Heather Erwin, Sarah Schreiber

**Affiliations:** Department of Kinesiology and Health Promotion, College of Education, University of Kentucky, Lexington, KY 40506, USA

**Keywords:** cognitive abilities, working memory, attention, impulsivity, aerobic, anaerobic

## Abstract

The present study examined the impact of aerobic and anaerobic exercise on cognitive abilities in eighth grade students at one middle school in southeastern US. It is known that youth do not meet recommended physical activity levels, yet there is research demonstrating a clear connection between aerobic exercise and academic performance. There is a gap in evidence regarding anaerobic exercise and cognitive capabilities. If professionals know which type of exercise is most beneficial for enhancing cognition in youth, it will behoove them to incorporate these practices in physical education and other areas throughout the school day for students. Thus, this research aimed to answer the following question: what is the effect of both aerobic and anaerobic exercise on the cognitive functions of eighth grade students, specifically measuring working memory, attention, and impulsivity? Fifty research participants (N = 31 males, 18 females, 1 other), aged 13–14, consented/assented to the complete study protocols. Participants engaged in an experiment containing three different cognitive tasks after partaking in three separate exercise conditions. The cognitive measures were the Stroop color–word task, the Flanker test, and the Go/No Go task, which all measured reaction time and the number of correct responses. The exercise conditions were baseline (after no exercise), aerobic (after a continuous running activity in PE), and anaerobic (after completing an interval dumbbell workout in PE). Each condition took place on a separate day to decrease the effect of confounding variables. The data were analyzed using the Scheffe modification of the MANOVA to determine whether multiple levels of the independent variable influenced the dependent variables (the reaction time and number of correct responses) or if a combination of variables influenced the outcomes. Each cognitive test was analyzed separately. The results showed that the exercise condition did significantly impact the reaction time and the number of correct responses for the Stroop color–word task [*F*(4, 254) = 8.59, *p* < 0.001; Pillai’s Trace = 0.24]. Specifically, aerobic exercise improved both reaction time (*p* < 0.001) and the number of correct responses (*p* = 0.001) compared to baseline, while anaerobic exercise improved just reaction time compared to baseline. To find out more detailed information about the specific dependent variable affected by the exercise program, individual ANOVA tests were conducted, resulting in statistical significance for both the number of correct responses and reaction time regarding the program. The partial Eta^2^ of 0.07 for the number of correct responses and 0.24 for reaction time indicated that 7% of the variance in correct responses and 24% of the variance in reaction time could be explained by the exercise program. While both are significant, reaction time is more impacted by exercise program than correct responses are. Given these findings, it is recommended that aerobic physical activity be offered in school prior to assigning cognitively challenging tasks, while quick anaerobic activity breaks should be used to refocus students’ attention. More research should be conducted to examine other cognitive abilities, as well as in other populations.

## 1. Introduction

During the COVID-19 pandemic, schools endured a huge shift from in-person learning to virtual learning. There were many negative impacts of this change on youth, one of which was a substantial reduction in physical activity for students. A meta-analysis published in the International Journal of Environmental Research and Public Health found an overall decrease in physical activity in children and adolescents during the pandemic. Specifically, the decline ranged from −10.8 min per day to −91 min per day [1]. Not only does decreased physical activity negatively impact children’s health, but previous research in the field shows clear evidence of the positive improvements that aerobic movement has on students in the classroom.

Aerobic exercise improves cognitive functions in three significant ways. First, exercise induces increased blood flow to the brain due to high oxygen demands, causing more nutrients to get efficiently delivered throughout the body to the organs [2]. The combination of these events allows more energy and oxygen to the brain, supporting better performance in cognitive tasks. Furthermore, exercise increases levels of brain-derived neurotrophic factor, BDNF, which helps promote brain plasticity, which is essential for learning and memory [3]. Lastly, exercise and physical activity generate chemical endorphins that serve as natural pain killers, thus decreasing stress levels [4]. The combination of these events allows more energy and oxygen to the brain, supporting better performance in cognitive tasks.

Research on aerobic-only movement breaks and academic performance has been positive in children, demonstrated by students who walked on a treadmill for 20 min (aerobic break) performing better on a Flanker task used to assess attention and inhibitory control, and on a standardized academic achievement test (reading, spelling, and math), than those who sat sedentary for the same time period [5]. In another study looking specifically at aerobic versus academic physical activity breaks during the school day, students who were provided with movement breaks with aerobic activity in mind showed marginally larger increases in reading achievement than their peers, who were given movement breaks with an academic content focus [6]. The aerobic movement breaks did not incorporate academic content, while the academic movement breaks integrated some academic content into the purpose of the physical activity.

Though research has shown a clear connection between the benefits of exercise and academic performance, most of the current literature focuses solely on aerobic exercise, thus leaving a gap regarding the comparison of anaerobic exercise and its effects on cognitive capabilities. Specifically, aerobic exercise refers to an activity that uses oxygen to produce energy; typically, this includes exercise that lasts over two minutes. For example, aerobic exercises typically done in physical education classes include running, bicycling, and jump roping. On the other hand, anaerobic exercise produces energy for the body without oxygen, and translates to physical activity of a shorter duration but higher intensity [7]. In physical education classes, anaerobic exercise can include sprinting short distances, weightlifting, and yoga. The importance of exercise for students, especially after the pandemic, in combination with the lack of knowledge comparing aerobic and anaerobic exercise in the field, defined the scope of this paper. Therefore, this research aimed to answer the following question: what is the effect of both aerobic and anaerobic exercise on cognitive functions in eighth grade students, specifically measuring working memory, attention, and impulsivity? The null hypothesis for all tests was that the dependent variable means were equal, despite changes in independent variables.

## 2. Materials and Methods

The research participants consisted of 51 8th grade students from a middle school in southeastern US. The students were from three different physical education classes at the middle school. They were selected because of the diverse sample they constituted: specifically, 33% Black, 24% White, 24% Mixed, and 30% other, or a more specific response (Table 1). The “Other” category primarily identified as Hispanic or Indian. It is important to note that the number of total participants varied throughout the study, due to students being absent, technology issues, or a lack of willingness. However, this did not affect the analysis, since only the means of each group were compared, as opposed to individual participant scores.

To analyze the impacts of aerobic and anaerobic exercise on various cognitive abilities, three peer-reviewed tests were conducted: the Stroop task, the Flanker test, and the Go/No Go task. The Stroop color–word task measures working memory and attention by requiring the participant to filter out conflicting information to correctly identify the color of the word [8]. For this test, participants are shown a series of color words (i.e., RED) printed in different colors (i.e., green). Their goal is to state the name of the color word (not the printed color). Their reaction times and number of correct responses helped measure the processing speed and interference. This is often influenced by self-regulatory control and executive attention.

The Flanker test is a set of multiple tests that examine selective attention and inhibition function [9]. For this test, participants are tasked with identifying the direction of a central arrow while discounting numerous contiguous arrows. Typically, as more random arrows are shown, the response time increases and accuracy decreases. This measures consciousness and attention.

Finally, the Go/No Go task measures correct responses to stimuli to assess impulsiveness [10]. Participants respond to the signals on the screen by pressing a button when they see the “Go” signal and refraining from pressing anything when they see the “No” signal. A key action that is measured is the ability to withhold a response, or inhibition, when seeing the “No” signal. All three tests measured two variables—reaction time and number of correct responses.

The three cognitive tests were combined through online experiment software that the participants completed on their own personal laptops, issued by the school. The questionnaire consisted of demographic survey questions at the beginning, then transitioned to the cognitive tests in the following order: the Stroop task, the Flanker test, and the Go/No Go task. In total, the time spent on the completion of the questionnaire ranged from 5 min to 9 min, with the average being 6.64 min. In order to assess the impact of different types of exercise on cognition, the experiment was administered three times under three separate conditions. The first condition was deemed the baseline, in which participants completed the questionnaire without having done any physical exercise in PE class, since it was the beginning of the semester. The teacher reviewed the syllabus, and then participants completed the questionnaire. The second condition, the aerobic condition, had the participants complete the questionnaire during the last ten minutes of PE class, after participating in a running tag game. The third condition, the anaerobic condition, had participants complete the questionnaire during the last ten minutes of PE class, after engaging in a weight resistance workout. Each condition took place on a separate day to decrease the effect of confounding variables.

Once the data from all three conditions were collected, they were analyzed using SPSS statistical analysis software. A multivariate analysis of variance, MANOVA, was conducted to determine whether multiple levels of the independent variable (condition) on their own or in combination with one another influenced the dependent variables by affecting the mean differences [11]. Each of the three cognitive tests (Stroop, Flanker, and Go/No Go) were analyzed separately to determine whether the various cognitive abilities tested were affected differently; therefore, a total of three MANOVAs were conducted. The Scheffe modification of the MANOVA was used because the number of data points for each condition was not equal. As mentioned previously, the two dependent variables measured were reaction time and the number of correct responses, while the independent variable was the condition, or exercise program, with the three different levels being baseline, aerobic, and anaerobic. The null hypothesis for all MANOVA tests was that dependent variable means were equal despite changes in independent variable level. There are numerous assumptions that should be met when using a MANOVA to ensure that the data set is appropriate for statical analysis. While these assumptions were all tested for, not all of them were approved for these data sets. Since not all the MANOVA assumptions were met, Pillai’s trace measure was used in the analysis. Pillai’s trace is the most reliable measure for small sample sizes and protects against type I errors that occur [12]. The limitations of not meeting all the assumptions will be explained further in the Conclusions Section of this research paper.

## 3. Results

Each cognitive assessment was analyzed separately; therefore, the results section will be divided into each respective test. The statistical significance, alpha, for all analysis tests was set at *p* ≤ 0.05.

### 3.1. The Stroop Color–Word Task

When comparing the mean values for the number of correct responses between exercise conditions, both the aerobic and anaerobic conditions had a higher mean than the baseline condition. There was a similar pattern for reaction time, as both aerobic and anaerobic conditions had lower mean reaction times than the baseline program. The aerobic program had the highest mean for the number of correct responses and lowest mean for reaction time (see Table 2).

When the mean differences were tested for significance through the MANOVA, the null hypothesis was rejected. In the Stroop test, the linear combination of reaction time and correct responses was significantly dependent on the exercise program (see Table 3; *p* < 0.001 for Pillai’s Trace program effect).

To find out more detailed information about the specific dependent variable that was affected by the exercise program, or if both were, individual ANOVA tests were performed. The results showed that there was statistical significance for both the number of correct responses and reaction time regarding the program source (see Table 4; # of correct responses: *p* = 0.001, reaction time: *p* < 0.001).

The partial eta squared of 0.070 for the number of correct responses and 0.237 for reaction time represents how 7% of the variance in correct responses and 23.7% of the variance in reaction time can be explained by the exercise condition. While both are significant, reaction time was more impacted by exercise condition than the number of correct responses was.

The last analysis for the Stroop test was used to determine which specific exercise condition impacted the different dependent variables using the multiple comparisons table. For number of correct responses, there was a statistically significant difference in means between the aerobic and baseline condition (see Table 5; *p* < 0.012). For reaction time, there was a statistically significant difference in means between both aerobic and baseline conditions, as well as anaerobic and baseline conditions (see Table 5; aerobic and baseline: *p* < 0.001, anaerobic and baseline: *p* < 0.001).

### 3.2. The Flanker Test

When comparing the mean values for the number of correct responses between exercise conditions, both the aerobic and anaerobic conditions had a slightly higher mean than the baseline condition. There was a similar pattern for reaction time, as both aerobic and anaerobic programs had lower mean reaction times than the baseline program. The aerobic condition had the highest mean for the number of correct responses, while the anaerobic program had the lowest mean for reaction time (see Table 6).

When the mean differences were tested for significance through the MANOVA, the null hypothesis failed to be rejected. In the Flanker Test, the sample did not provide sufficient evidence to conclude that the linear combination of reaction time and correct responses were significantly dependent on the exercise condition (see Table 7; *p* = 0.124 for Pillai’s trace program effect).

To determine if only one or more dependent variables were affected by the exercise condition, individual ANOVA tests were required. The results showed that there is no statistical significance for either the number of correct responses or reaction time regarding the program source (see Table 8; # of correct responses: *p* = 0.182, reaction time: *p* = 0.061).

The last analysis for the Flanker Test was used to further validate that the specific exercise condition had no significant impact on the various dependent variables using the multiple comparisons table. For the number of correct responses, there was again no statistically significant difference in means between any exercise condition (see Table 9; aerobic and baseline: *p* = 0.197, anaerobic and baseline: *p* = 0.506). For reaction time, there was also no statistically significant difference in means between any exercise condition (see Table 9; aerobic and baseline: *p* = 0.219, anaerobic and baseline: *p* = 0.085).

### 3.3. The Go/No Go Task

When comparing the mean values for reaction time between exercise conditions, both the aerobic and anaerobic conditions had a slightly lower mean than the baseline condition. The overall lowest reaction time was in the anaerobic condition. There was no difference between any exercise conditions for the number of correct responses (see Table 10).

When the mean differences were tested for significance through the MANOVA, the null hypothesis failed to be rejected. In the Go/No Go task, the sample did not provide sufficient evidence to conclude that the linear combination of reaction time and correct responses was significantly dependent on the exercise condition (see Table 11; *p* = 0.925 for Pillai’s trace program effect).

To determine if one or more dependent variables were affected by the exercise condition, individual ANOVA tests were conducted. The results showed that there was no statistical significance for either the number of correct responses or reaction time regarding the program source (see Table 12; reaction time: *p* = 0.695, # of correct responses: *p* = 0.902).

The last analysis for the Go/No Go task was used to further validate that the specific exercise condition had no significant impact on the various dependent variables using the multiple comparisons table. For reaction time, there was also no statistically significant difference in means between any exercise conditions (see Table 13; aerobic and baseline: *p* = 0.814, anaerobic and baseline: *p* = 0.729). For the number of correct responses, there was again no statistically significant difference in means between any exercise conditions (see Table 13; aerobic and baseline: *p* = 0.907, anaerobic and baseline: *p* = 0.995).

## 4. Conclusions

This study answered the following research question: “What is the effect of both aerobic and anaerobic exercise on cognitive functions in 8th grade students, specifically measuring working memory, attention, and impulsivity”? The Stroop color and word task was the only test that had statistically significant results regarding the effect of exercise type on cognitive function, specifically regarding reaction time and the number of correct responses. In terms of the comparison between aerobic and anaerobic exercise, the multiple comparisons table showed that only aerobic exercise improved the number of correct answers, while both forms of exercise improved the reaction time. Therefore, it can be concluded that while both forms of exercise produced positive impacts on working memory and attention, aerobic exercise had a greater effect in general.

The measurements of the Stroop task, working memory and attention, can be aligned with the number of correct responses and reaction time, respectively. Since correctly identifying the color of the word is a more difficult task than simply responding quickly, it can be said that aerobic exercise improved responses to more challenging cognitive tasks, while anaerobic exercise improved students’ attention. This can be translated to benefit the classroom setting in multiple different ways. Teachers can conduct physical activity breaks throughout the school day based on upcoming goals and tasks. For instance, if students seem to be tired or disinterested, an anaerobic exercise like jumping jacks can quickly improve students’ attention by getting blood flow moving back through organs, and specifically the brain. However, if they are about to work on a task that is more cognitively challenging and requires working memory, they should implement a longer aerobic exercise break like a walk or light jog around the building to benefit their academic performance. Furthermore, physical education teachers and core subject teachers can collaborate to ensure students are prepared for various cognitive tests. Administrators can work to plan schedules so that students have adequate opportunities for aerobic and anaerobic activity before working on different types of academic tasks, projects, tests, and assessments.

The Go/No Go task had the least significant results and differences between means for both the number of correct responses and reaction time. This likely can be explained by the simplicity of the test. Students were instructed to press the space bar if the oval on the screen was green or do nothing (refrain) if the oval was red. The scores were relatively high for the baseline condition, and there was not much room for improvement based on the type of exercise condition. It can be concluded that basic cognitive functions do not improve in a similar pattern to more complex skills. This concept contradicts the current research findings regarding the positive impact of exercise at managing impulsivity in children with ADHD [13]. However, these research findings did not initially determine if any participants had an ADHD diagnosis or symptoms.

There are a few limitations to this research study that are important to discuss. First and foremost, the sample was relatively small and only included eighth grade students from one middle school in the southeast. Therefore, the results cannot be generalized to many different groups of children or adolescents. More research should be conducted on different populations to compare findings. There was also a possibility of testing familiarity for the participants, since they completed the questionnaire three times following the different conditions. It is possible that the students had improved scores on the aerobic and anaerobic programs because they already had practice taking the cognitive tests. Future research should randomize the order of conditions and testing. Lastly, not all statistical assumptions were met because it was difficult to acquire a perfect data set for the MANOVA. Despite this, it was still appropriate to run the MANOVA with this potential limitation.

Not only do the findings of this study support the notion that physical activity breaks and exercise during school promotes cognitive functioning, they offer more specific suggestions for the type of physical activity and movement to offer students for future interventions. Most notably, future research should aim to further understand the relationship between aerobic and anaerobic exercise and how each impact our cognitive functions differently. There is a vast number of cognitive abilities, such as long-term memory, problem solving, and decision making, among others, and each may exhibit different responses following various types of exercise. It would be beneficial for educators, students, parents, and school board members to assess how physical activity of varying intensities and types impacts each of these cognitive skills to maximize student success.

## Figures and Tables

**Table 1:** Participant demographics.

Category	Sub-Category	Frequency (N)	Percentage (%)
Age	13	35	70
	14	15	30
Gender	Male	31	62
	Female	18	36
	Other	1	2
Ethnicity	Black	11	22
	White	12	24
	Mixed	12	24
	Other or more specific	15	30

**Table 2:** Descriptive statistics.

	Program	Mean	Std. Deviation	N
#Correct	Aerobic	26.62	6.44	39
	Anaerobic	34.50	8.20	44
	Baseline	30.57	11.77	47
	Total	33.72	9.51	130
Reaction Time	Aerobic	970.15	165.36	39
	Anaerobic	1034.47	210.17	44
	Baseline	1237.61	238.27	47
	Total	1088.61	237.39	130

**Table 3:** Multivariate test for Stroop test.

Effect		Value	F	Hypothesis df	Error df	Sig.
Intercept	Pillai’s Trace	0.99	5312.03	2.00	126.00	<0.001
	Wilks’ Lambda	0.01	5312.03	2.00	126.00	<0.001
	Baseline	84.32	5312.03	2.00	126.00	<0.001
	Total	84.32	5312.03	2.00	126.00	<0.001
Program	Pillai’s Trace	0.23	8.59	4.00	254.00	<0.001
	Wilks’ Lambda	0.76	9.17	4.00	252.00	<0.001
	Baseline	0.31	9.74	4.00	250.00	<0.001
	Total	0.31	19.71	2.00	127.00	<0.001

**Table 4:** Between-subject ANOVA for Stroop test.

Source	Dependent Variable	Sig.	Partial Eta Squared	Noncent. Parameter	Observed Power ^a^
Corrected Model	#Correct	0.01	0.07	9.59	0.79
	Reaction Time	<0.001	0.24	39.35	1.00
Intercept	#Correct	<0.001	0.93	1739.29	1.00
	Reaction Time	<0.001	0.96	3453.92	1.00
Program	#Correct	0.01	0.07	9.59	0.79
	Reaction Time	<0.001	0.24	39.35	1.00
Error	#Correct				
	Reaction Time				
Total	#Correct				
	Reaction Time				
Corrected Total	#Correct				
	Reaction Time				

^a^ Computed using alpha = 0.05.

**Table 5:** Multiple comparisons for Stroop test.

Dependent Variable	(I) Program	(J) Program	Mean Difference (I–J)	Std. Error	Sig.
#Correct	Aerobic	Anaerobic	2.12	2.03	0.583
		Baseline	6.04	2.00	0.012
	Anaerobic	Aerobic	−2.12	2.03	0.583
		Baseline	3.93	1.94	0.133
	Baseline	Aerobic	−6.04	2.00	0.02
		Anaerobic	−3.93	1.94	0.133
Reaction Time	Aerobic	Anaerobic	−64.32	45.98	0.379
		Baseline	−267.46	45.28	<0.001
	Anaerobic	Aerobic	64.32	45.98	0.379
		Baseline	−203.14	43.85	<0.001
	Baseline	Aerobic	267.46	45.28	<0.001
		Anaerobic	203.14	43.85	<0.001

**Table 6:** Descriptive statistics for Flanker test.

	Program	Mean	Std. Deviation	N
#Correct	Aerobic	35.51	12.04	37
	Anaerobic	34.03	11.48	29
	Baseline	30.75	10.98	40
	Total	33.31	11.57	106
Reaction Time	Aerobic	803.85	362.63	37
	Anaerobic	756.33	305.01	29
	Baseline	932.95	294.23	40
	Total	839.56	328.23	106

**Table 7:** Multivariate test for Flanker test.

Effect		Value	F	Hypothesis df	Error df	Sig.
Intercept	Pillai’s Trace	0.96	1163.44	2.00	102.00	<0.001
	Wilks’ Lambda	0.04	1163.44	2.00	102.00	<0.001
	Hotelling’s Trace	22.81	1163.44	2.00	102.00	<0.001
	Roy’s Largest Root	22.81	1163.44	2.00	102.00	<0.001
Program	Pillai’s Trace	0.07	1.83	4.00	206.00	0.124
	Wilks’ Lambda	0.93	1.83	4.00	204.00	0.124
	Hotelling’s Trace	0.07	1.83	4.00	202.00	0.124
	Roy’s Largest Root	0.06	3.30	2.00	103.00	0.041

**Table 8:** Between-subjects ANOVA for Flanker test.

Source	Dependent Variable	Sig.	Partial Eta Squared	Noncent. Parameter	Observed Power ^a^
Corrected Model	#Correct	0.18	0.03	3.46	0.36
	Reaction Time	0.06	0.05	5.73	0.55
Intercept	#Correct	<0.001	0.90	880.31	1.00
	Reaction Time	<0.001	0.87	690.64	1.00
Program	#Correct	0.18	0.03	3.46	0.36
	Reaction Time	0.06	0.05	5.74	0.55
Error	#Correct				
	Reaction Time				
Total	#Correct				
	Reaction Time				
Corrected Total	#Correct				
	Reaction Time				

^a^ Computed using alpha = 0.05.

**Table 9:** Multiple comparisons for Flanker test.

Dependent Variable	(I) Program	(J) Program	Mean Difference (I–J)	Std. Error	Sig.
#Correct	Aerobic	Anaerobic	1.48	2.85	0.874
		Baseline	4.76	2.62	0.197
	Anaerobic	Aerobic	−1.48	2.85	0.874
		Baseline	3.28	2.80	0.506
	Baseline	Aerobic	−4.76	2.62	0.197
		Anaerobic	−3.28	2.80	0.506
Reaction Time	Aerobic	Anaerobic	47.51	79.99	0.839
		Baseline	−129.10	73.57	0.219
	Anaerobic	Aerobic	−47.51	79.99	0.839
		Baseline	−176.61	78.66	0.085
	Baseline	Aerobic	129.10	73.57	0.219
		Anaerobic	176.61	78.66	0.085

**Table 10:** Descriptive statistics for Go/No Go test.

	Program	Mean	Std. Deviation	N
#Correct	Aerobic	381.90	82.29	37
	Anaerobic	377.99	90.12	29
	Baseline	394.01	73.83	40
	Total	385.23	81.11	106
Reaction Time	Aerobic	23.64	2.62	37
	Anaerobic	23.79	1.35	29
	Baseline	23.84	1.35	40
	Total	23.76	1.88	106

**Table 11:** Multivariate test for Go/No Go test.

Effect		Value	F	Hypothesis df	Error df	Sig.
Intercept	Pillai’s Trace	0.99	7943.74	2.00	100.00	<0.001
	Wilks’ Lambda	0.01	7943.74	2.00	100.00	<0.001
	Hotelling’s Trace	158.88	7943.74	2.00	100.00	<0.001
	Roy’s Largest Root	158.88	7943.74	2.00	100.00	<0.001
Program	Pillai’s Trace	0.01	0.22	4.00	202.00	0.925
	Wilks’ Lambda	0.99	0.22	4.00	200.00	0.926
	Hotelling’s Trace	0.01	0.22	4.00	198.00	0.927
	Roy’s Largest Root	0.01	0.37	2.00	101.00	0.694

**Table 12:** Between-subject ANOVA for Go/No Go test.

Source	Dependent Variable	Sig.	Partial Eta Squared	Noncent. Parameter	Observed Power ^a^
Corrected Model	#Correct	0.70	0.01	0.73	0.11
	Reaction Time	0.90	0.00	0.21	0.07
Intercept	#Correct	<0.001	0.96	2275.78	1.00
	Reaction Time	<0.001	0.99	16,043.49	1.00
Program	#Correct	0.70	0.01	0.73	0.11
	Reaction Time	0.90	0.00	0.21	0.07
Error	#Correct				
	Reaction Time				
Total	#Correct				
	Reaction Time				
Corrected Total	#Correct				
	Reaction Time				

^a^ Computed using alpha = 0.05.

**Table 13:** Multiple comparisons for Go/No Go test.

Dependent Variable	(I) Program	(J) Program	Mean Difference (I–J)	Std. Error	Sig.
#Correct	Aerobic	Anaerobic	3.91	20.24	0.982
		Baseline	−12.12	18.85	0.814
	Anaerobic	Aerobic	−3.91	20.24	0.982
		Baseline	−16.03	20.13	0.729
	Baseline	Aerobic	12.12	18.85	0.814
		Anaerobic	16.03	20.13	0.729
Reaction Time	Aerobic	Anaerobic	−0.14	0.47	0.954
		Baseline	−0.19	0.44	0.907
	Anaerobic	Aerobic	0.14	0.47	0.954
		Baseline	−0.05	0.47	0.995
	Baseline	Aerobic	0.19	0.44	0.907
		Anaerobic	0.05	0.47	0.995

## Data Availability

Data can be requested upon permission from the first author.

## References

[1] Rossi L., Behme N., Breuer C. (2021). Physical activity of children and adolescents during the COVID-19 pandemic—A scoping review. Int. J. Environ. Res. Public Health.

[2] McGregor G. (2021). How Exercise Affects the Brain. Life Sciences. https://lifesciences.byu.edu/how-exercise-affects-your-brain.

[3] Bathina S., Das U.N. (2015). Brain-derived neurotrophic factor and its clinical implications. Arch. Med. Sci..

[4] Anxiety and Depression Association of America (2022). Physical Activity Reduces Stress. https://adaa.org/understanding-anxiety/related-illnesses/other-related-conditions/stress/physical-activity-reduces-st.

[5] Hillman C.H., Pontifex M.B., Raine L.B., Castelli D.M., Hall E.E., Kramer A.F. (2009). The effect of acute treadmill walking on cognitive control and academic achievement in preadolescent children. Neuroscience.

[6] Fedewa A.L., Fettrow E., Erwin H., Ahn S., Farook M. (2018). Academic-based and aerobic-only movement breaks: Are there differential effects on physical activity and achievement?. Res. Q. Exerc. Sport.

[7] Patel H., Alkhawam H., Madanieh R., Shah N., Kosmas C.E., Vittorio T.J. (2017). Aerobic vs anaerobic exercise training effects on the cardiovascular system. World J. Cardiol..

[8] University of Utah (2020). Stroop Test. Genetic Science Learning Center. https://teach.genetics.utah.edu/content/memory/Stroop-test.pdf.

[9] Eriksen Flanker Task ScienceDirect Topics. https://www.sciencedirect.com/topics/neuroscience/eriksen-flanker-task.

[10] (2021). Go/No-Go Task. PsyToolKit. https://www.psytoolkit.org/experiment-library/go-no-go.html.

[11] Laerd Statistics One-Way Manova in SPSS Statistics. One-Way MANOVA in SPSS Statistics—Step-by-Step Procedure with Screenshots. https://statistics.laerd.com/spss-tutorials/one-way-manova-using-spss-statistics.php.

[12] Ateş C., Kaymaz Ö., Kale H.E., Tekindal M.A. (2019). Comparison of test statistics of nonnormal and unbalanced samples for multivariate analysis of variance in terms of type-I error rates. Comput. Math. Methods Med..

[13] Cerrillo-Urbina A.J., García-Hermoso A., Sánchez-López M., Pardo-Guijarro M.J., Santos Gómez J.L., Martínez-Vizcaíno V. (2015). The effects of physical exercise in children with attention deficit hyperactivity disorder: A systematic review and meta-analysis of randomized control trials. Child Care Health Dev..

